# Clinical Innovation Poster Abstracts

**DOI:** 10.1080/24740527.2017.1329312

**Published:** 2017-05-22

## The Queen’s University Hotel Dieu Hospital Chronic Pain Clinic, an evolution

Elizabeth Brown^a^, Scott Duggan^b^, and Peter Ellis^c^

^a^Chronic Pain Clinic, Hotel Dieu Hospital, Kingston, ON, Canada; ^b^Anaesthesiology and Perioperative Medicine, Queen’s University, Kingston, ON, Canada; ^c^Surgery, Queen’s University, Kingston, ON, Canada

**CONTACT** Elizabeth Brown browne2@hdh.kari.net

© 2017 Elizabeth Brown, Scott Duggan, and Peter Ellis. Published with license by Taylor & Francis.

This is an Open Access article distributed under the terms of the Creative Commons Attribution License (http://creativecommons.org/licenses/by/3.0/), which permits unrestricted use, distribution, and reproduction in any medium, provided the original work is properly cited. The moral rights of the named author(s) have been asserted.

Since its inception in 2011, the Queen’s University Hotel Dieu Hospital Chronic Pain Clinic has evolved from; one physician, two half-time nurses, two days and fifty patient encounters per week, one half-time support staff and an average waiting list of four years, to a six clinician, five nurse, five days and three hundred patient encounters per week, 2.5 support staff, 1.5 physiotherapists, two half-time occupational therapists, one social worker and one psychologist. Our average waiting list is now about eight months with an aim to achieve less than six months. Formerly, blind trigger point muscle injections were the only procedures offered, and now these, as well as ultrasound and image-intensifier guided ESI, joint and nerve block procedures occur weekly. Lidocaine and Magnesium infusions are also provided.

This broadening and deepening of services was made possible through a multi-year capital and operating grant from the Ontario Ministry of Health.

Independent of this budgetary windfall, the increase in clinic efficiencies is primarily due to the implementation of up-front informational and educational sessions with our allied health professionals, mandated geographical referral boundaries, and rigorous and timely intake triaging. Most referrals come from speciality services, regional emergency rooms and primary care teams. Triaging now occurs within ten days of request, and involves consideration of severity, location, cause and description of pain, associated diagnoses, medications, functional limitations and social supports.

## PACE IT: a creative, flexible, interprofessional model for rural and remote access

Jennifer Cano, Andrew Koscielniak, and Matthew Schmidt

Chronic Pain Management Program, St. Joseph’s Health Centre, Thunder Bay, ON, Canada

© 2017 Jennifer Cano, Andrew Koscielniak, and Matthew Schmidt. Published with license by Taylor & Francis.

This is an Open Access article distributed under the terms of the Creative Commons Attribution License (http://creativecommons.org/licenses/by/4.0/), which permits unrestricted use, distribution, and reproduction in any medium, provided the original work is properly cited.

The **P**ain **A**ssessment, **C**ollaborative **E**ducation and **I**nterprofessional **T**herapy (PACE IT) program provides services to clients with chronic pain living in northwestern Ontario – a region roughly the size of France with a large Indigenous population. PACE IT allows our team to be creative and flexible in the delivery of treatment to best meet client needs. Many of the clients who are referred to PACEIT cannot tolerate the intensive six-week program or are unable to attend for a variety of socio-economic reasons (e.g. work, childcare). The program encourages self management and is comprised of 16 educational and gentle movement sessions delivered in-person once weekly or via teleconference directly to clients’ communities (e.g., home, nursing station, school). It is also possible to group the sessions for clients able to travel to our location in a large urban centre (Thunder Bay) for three to five days. Clients can attend individual appointments (via OTN, telephone or in person) with members from our interprofessional team as appropriate. Outcome measures are administered pre-intervention and three months and six months after completing the sessions. Fitness measures are administered regularly and results are shared with clients to provide motivation. We are partnering with local and regional recreation/fitness facilities to advocate for rates, programming and support appropriate for clients with chronic pain. As a result clients are introduced to resources that provide lifelong opportunities to develop and maintain physical fitness and manage pain.

## An internet-based coping skills intervention for psychosocial problems after a distal radius fracture

Folarin Babatunde^a^, Joy MacDermid^a,b,c^, Ruby Grewal^b,c^, and Luciana Macedo^a^

^a^School of Rehabilitation Science, McMaster University, Hamilton, Ontario, Canada; ^b^Hand and Upper Limb Centre, St Josephs Hospital, London, Ontario, Canada; ^c^University of Western Ontario, London, Ontario, Canada

**CONTACT** Folarin Babatunde babatufo@mcmaster.ca

© 2017 Folarin Babatunde, Joy MacDermid, Ruby Grewal, and Luciana Macedo. Published with license by Taylor & Francis.

This is an Open Access article distributed under the terms of the Creative Commons Attribution License (http://creativecommons.org/licenses/by/3.0/), which permits unrestricted use, distribution, and reproduction in any medium, provided the original work is properly cited. The moral rights of the named author(s) have been asserted.

Introduction: Distal radius fracture (DRF) is the most common upper-extremity problem encountered in emergency orthopedic care. Although, many patients recover well after one year, up to 30% and 30% of individuals with DRF continue to have prolonged pain and disability respectively. There has been growing recognition of psychosocial risk factors such as catastrophizing, pain anxiety, low mood and fear of movement as predictors of long-term pain and disability after a DRF.

Program Objectives: The hand therapy coping skills program was designed to address psychosocial risk factors after a DRF.

Target Population: Canadian adults (>18 years) diagnosed with a DRF and with a score of ≤4 on the Pain Belief Screening Instrument.

Program Description: The program consists of 5 coping skills modules, homework tasks, additional resources and weekly e-mail from a researcher. Module 1 provides education using Therapeutic Neuroscience Education principles. Module 2 teaches skills on managing thoughts based on cognitive behavioral therapy principles. Module 3 teaches skills on managing daily activity using pacing and scheduling principles. Module 4 teaches skills in identifying and overcoming barriers to treatment adherence. Module 5 teaches relaxation techniques. Each module takes about 30 minutes and accessible though www.handtherapycopingskill.com.

Future Directions: A feasibility and pilot study is currently underway to evaluate the feasibility, acceptability, credibility of the program and potential impacts of the program on pain, disability and anxiety/depressive symptoms of Canadian adults with DRF.

## Improving patient expectations of chronic pain management

Joanne Stewart, Ian Beauprie, Patty Livingston, Krista Farr, Jessica Howe, and Darlene Davis

Department of Anesthesia, Pain Management & Perioperative Medicine, Nova Scotia Health Authority, Halifax, Nova Scotia, Canada

**CONTACT** Joanne Stewart joanne.stewart@nshealth.ca

© 2017 Joanne Stewart, Ian Beauprie, Patty Livingston, Krista Farr, Jessica Howe, and Darlene Davis. Published with license by Taylor & Francis.

This is an Open Access article distributed under the terms of the Creative Commons Attribution License (http://creativecommons.org/licenses/by/3.0/), which permits unrestricted use, distribution, and reproduction in any medium, provided the original work is properly cited. The moral rights of the named author(s) have been asserted.

Introduction/Aim: All new patients referred to the Nova Scotia Health Authority (NSHA) pain clinics were required to attend a mandatory group medical visit (GMV). These sessions provided basic information on the complexities of chronic pain, pain treatments, pain self management programs, and an overview of pain management services available. The goals were to decrease wait times, decrease new consult “no show” rates, and to promote patient active participation in their pain management strategies.

Methods: GMVs were facilitated by a pain specialist physician, pain clinic RN, and a psychologist/physiotherapist. A one hour semi interactive method of teaching was utilized. Appointment letters were sent to the referring clinician and patient. Non-attendee referrals were returned to referring clinician. Registration and evaluation forms were filled out by patients after each session including an indicator that the patient still wanted to be seen. Ethics committee approval was not required for the initiation or evaluation of this program (project considered part of ongoing QA initiatives).

Results: Overall patient feedback was encouraging. Adjustment to present style, content, and core messaging was implemented. All pain clinic physicians will rotate through two GMVs bi-monthly. Results of twenty GMVs will be presented at the CPS meeting 2017.

Discussion/Conclusion: Evaluation will include patient feedback, tracking patterns and characteristics of no show appointments, and participation in pain self-management programs. Further analysis and follow up will be needed in order to determine impact on patient wait list.

## Comprehensive acute pain education at a tri-site organization

Carrie Branch^a^, Danielle Dunwoody^a^, and Julie Mcbrien^b^

^a^Acute Pain Service, Halton Healthcare, Oakville, ON, Canada; ^b^Program Director Surgery, Halton Healthcare, Oakville, ON, Canada

**CONTACT** Carrie Branch cbranch@haltonhealthcare.com

© 2017 Carrie Branch, Danielle Dunwoody, and Julie Mcbrien. Published with license by Taylor & Francis.

This is an Open Access article distributed under the terms of the Creative Commons Attribution License (http://creativecommons.org/licenses/by/3.0/), which permits unrestricted use, distribution, and reproduction in any medium, provided the original work is properly cited. The moral rights of the named author(s) have been asserted.

Pain education to new nursing staff has historically been a standard for Halton Healthcare. A needs assessment was carried out to examine how the current structure of the education met the knowledge gaps of the nursing staff. The assessment revealed a significant need for standardized education that was consistenly available across the three community hospitals within the organization.

Based on the themes identified in the needs assessment from staff, physicians and the incident reporting system, a comprehensive half day workshop was offered to nurses from all sites, monthly. Workshop curriculum was developed based on CNO and RNAO standards and included: the pain pathway, multimodal analgesia, pharmacology, pump training and advanced pain management techniques.

Outcome measures included a 3-point narrative scale and open-ended questions. Curricula were modified to meet the needs voiced in the evaluations and barriers were identified. Challenges in the execution of the workshop included: funding, wide variations in baseline pain knowledge of learners, and developing the content further to meet the needs of multiple patient care areas.

Within a 5 month period and with 4 workshops conducted, the target audience was reached and the workshop was acknowledged as a valuable source of education within the organization. Standardization of pain education will ensure the learning needs of the staff are met, increase consistency in care and contribute to exemplary patient care.

## Brief education videos for healthcare providers on pain assessment and management in older adults: Development and preliminary qualitative evaluation

Susan M. Tupper^a^, Kirstian Gibson^b^, Julia E. Bareham^c^, Tracy L. Danylyshen-Laycock^d^, and Anita Bergen^d^

^a^Pain Management, Saskatoon Health Region, Saskatoon, SK, Canada; ^b^Psychology, University of Saskatchewan, Saskatoon, SK, Canada; ^c^Prescription Review Program, College of Physicians and Surgeon of Saskatchewan, Saskatoon, SK, Canada; ^d^Seniors’ Health and Continuing Care, Saskatoon Health Region, Saskatoon, SK, Canada

**CONTACT** Susan M. Tupper susan.tupper@usask.ca

© 2017 Susan M. Tupper, Kirstian Gibson, Julia E. Bareham, Tracy L. Danylyshen-Laycock, and Anita Bergen. Published with license by Taylor & Francis.

This is an Open Access article distributed under the terms of the Creative Commons Attribution License (http://creativecommons.org/licenses/by/3.0/), which permits unrestricted use, distribution, and reproduction in any medium, provided the original work is properly cited. The moral rights of the named author(s) have been asserted.

Aims: Evaluate nursing perspectives on a new series of brief education videos on pain in older adults.

Background: Pain in older adults is often under-recognized and under-managed. Nurse educators in the Saskatoon Health Region requested education materials for use in team huddles or education days to prompt best practice for pain assessment and management in older adults. A series of 13 brief videos was developed.

Development: Interviews with Seniors’ Health administrative leaders, clinical nurse educators, and interprofessional practice leads provided the needs assessment foundation for video content. A core development team reviewed best-practice literature and wrote the video script. Feedback was provided on the script by an interprofessional review team which included two patient/family advocates. Videos were professionally filmed and edited.

Qualitative Evaluation Methods: A 120-minute focus group was held with nine nursing staff from a single long-term care home in the Saskatoon Health Region. Participants viewed six videos and responded to facilitating questions. The focus group was audio-recorded, transcribed verbatim, and analyzed with an Interpretive Description methodology by two independent coders.

Results: Major coded themes were: challenges for obtaining/maintaining pain knowledge, benefits/requirements of pain education, congruence and integration with prior knowledge, pain education/training sources, and barriers to pain assessment/management.

Conclusions: Preliminary findings demonstrate perceived acceptability and clinical utility of a series of brief education videos on pain assessment and management in older adults for nursing staff from a single long-term care home. Further research is needed to determine the effect of the videos on pain knowledge and practice.

## Opioid Education Partnership: Pilot test results for an interdisciplinary education initiative targeting prescription opioid misuse

Feng Chang^a,b^, Heba Tallah Mohammed^a^, Agnes Kluz^b^, Tejal Patel^a^, and Rosemary Killeen^a^

^a^School of Pharmacy, University of Waterloo, Waterloo, Ontario, Canada; ^b^Gateway Centre of Excellence in Rural Health, Goderich, Ontario, Canada

**CONTACT** Feng Chang feng.chang@uwaterloo.ca

© 2017 Feng Chang, Heba Tallah Mohammed, Agnes Kluz, Tejal Patel, and Rosemary Killeen. Published with license by Taylor & Francis.

This is an Open Access article distributed under the terms of the Creative Commons Attribution License (http://creativecommons.org/licenses/by/3.0/), which permits unrestricted use, distribution, and reproduction in any medium, provided the original work is properly cited. The moral rights of the named author(s) have been asserted.

The Opioid Education Partnership is a Health Canada-funded interdisciplinary initiative that developed and tested a pilot education program, consisting of 8 interactive online modules, designed to help physicians, pharmacists, students and trainees enhance understanding of prescription opioid use and promote collaborative management. The program is accredited by the College of Family Physicians of Canada.

Recruitment took place from May to September 2016 followed by pilot testing from September 2016 to March 2017. Displays at three national professional conferences invited interested clinicians to participate. Eligible participants in medicine and pharmacy were provided free access to the online program and completed the program at their own pace. Evaluation consisted of a baseline survey before program initiation, satisfaction questionnaire at completion, and follow-up survey 6 weeks after. The surveys addressed perceptions and self-confidence towards prescription opioid management, and the program’s perceived impact on practice.

A total of 274 eligible participants registered, 189 (69%) completed the baseline survey and 43 (15.7%) completed all 8 modules. Website traffic data showed an average of 21,250 hits per month (9,674 - 45,730), mostly from Canada and the US, but also from other parts of the world including Europe, Asia, Africa and Australia. Interim analysis showed participants rated the program as useful (97.8%), increased their knowledge (88.9%) and felt the content was at the right level (68%). A majority (68.1%) expected to makes some to significant changes in their practice as a result. Most (86.7%) would strongly recommend the program to their peers.

## The mind-body connection program for somatization in youth: A novel intervention for engaging families in mental health care

Amrit Dhariwal^a^, Andrea Chapman^a^, and Theresa Newlove^b^

^a^Psychiatry, BC Children’s Hospital, Vancouver, BC, Canada; ^b^Psychology, BC Children’s Hospital, Vancouver, BC, Canada

**CONTACT** Amrit Dhariwal amrit.dhariwal@cw.bc.ca

© 2017 Amrit Dhariwal, Andrea Chapman, and Theresa Newlove. Published with license by Taylor & Francis.

This is an Open Access article distributed under the terms of the Creative Commons Attribution License (http://creativecommons.org/licenses/by/4.0/), which permits unrestricted use, distribution, and reproduction in any medium, provided the original work is properly cited.

Somatization disorders are mental health conditions that present as disabling and persistent physical symptoms. where no medical cause can be found (e.g., pain, neurological symptoms). They affect up to 13% of youth and can severely impair everyday functioning. Early treatment is needed to prevent continuance into adulthood.

The way we feel (emotion) is often experienced physically. When emotions become overwhelming and go chronically unrecognized, serious physical symptoms can emerge or worsen over time. Yet those suffering from somatization conditions tend to distrust emotional explanations, fearing a medical diagnosis has been missed, and often find themselves in a cycle of increasing medical tests and treatments, treatment failure, unwanted side effects, and suffering. In parallel, psychological approaches can also overlook emotions and focus on behavioural symptom management, with only limited benefit. To provide better relief, we propose an innovative approach that will help youth and families develop confidence in their somatization diagnosis and target underlying emotions. In our Mind Body Connection program, clinicians listen to and validate the patient’s medical explanation, provide a non-judgmental and scientific understanding of how emotions are expressed in the body, and support families to respond to and manage overwhelming emotions in order to reduce physical ailments.

Our program has been piloted in a sample of 46 youth (ages 13-17) with diverse symptom presentations. On empirically validated self- and caregiver-report pre/post measures, the program is correlated with improvements in symptoms, functioning, acceptance of psychological, and decreased medical visits. A controlled study for further evaluation is planned.

## Something old, something new: Innovation and best practice in complex pain management

Sarah Keefe^a^ and Michael Sangster^b^

^a^Occupational Therapy, IWK Health Centre, Halifax, Nova Scotia, Canada; ^b^Physiotherapy, IWK Health Centre, Halifax, Nova Scotia, Canada

**CONTACT** Sarah Keefe sarah.keefe@iwk.nshealth.ca

© 2017 Sarah Keefe and Michael Sangster. Published with license by Taylor & Francis.

This is an Open Access article distributed under the terms of the Creative Commons Attribution License (http://creativecommons.org/licenses/by/4.0/), which permits unrestricted use, distribution, and reproduction in any medium, provided the original work is properly cited.

Introduction: Current evidence supports multidisciplinary management of adolescents with complex pain syndromes to remediate pain and improve daily function. Advances in our understanding of complex pain suggest that maladaptive cortical change correlates with pain presentation. Emerging brain-based interventions that target maladaptive neuroplasticity combined with traditional occupation-based interventions may offer an effective management approach.

Objectives: To describe a management approach combining interventions targeting somatosensory and motor areas of the brain with traditional goal setting and use of meaningful occupations to improve daily function via a case presentation of a 13 year-old female with complex unilateral wrist pain experiencing loss of function in all areas.

Methods: Occupational performance problems were identified using semi-structured interview and the Canadian Occupational Performance Measure (COPM). Goal setting was completed with client identifying goals related to gradually resuming meaningful occupations using a progressive pacing strategy. Education regarding pacing was provided and client was encouraged to reflect upon weekly performance of meaningful occupations during regular check-ins to monitor goal attainment. Client additionally participated in a Graded Motor Imagery therapeutic approach consisting of implicit motor imagery using Recognise app and mirror therapy.

Results: Positive change scores in performance (6.75) and satisfaction (7.25) were calculated. Client reports she continues to experience pain but lower levels and reduced duration with increased confidence in managing flares of pain while maintaining function using pacing strategies.

Conclusions: This case highlights the effective combination of traditional occupational therapy interventions with emerging therapies that target the brain to decrease pain and improve occupational performance.

## Left or right? Some direction for the management of complex regional pain syndrome

Michael Sangster^a^, Ayala Y. Gorodzinsky^b^, Kathryn A. Birnie^c^, Kristin Taylor^a^, Stuart Wright^d^, Shelley Lowther^e^, Allen Finley^f^, and Nichole Helme

^a^Physiotherapy, IWK Health Centre, Halifax, Nova Scotia, Canada; ^b^Psychology, IWK Health Centre, Halifax, Nova Scotia, Canada; ^c^Lawrence S. Bloomberg Faculty of Nursing, University of Toronto, Toronto, Ontario, Canada; ^d^Pediatric Anesthesia, Dalhousie University, Halifax, Nova Scotia, Canada; ^e^Pediatric Pain Management, IWK Health Centre, Halifax, Nova Scotia, Canada; ^f^Anesthesia & Psychology, Dalhousie University, Halifax, Nova Scotia, Canada

**CONTACT** Michael Sangster Michael.sangster@iwk.nshealth.ca

© 2017 Michael Sangster, Ayala Y. Gorodzinsky, Kathryn A. Birnie, Kristin Taylor, Stuart Wright, Shelley Lowther, Allen Finley, and Nichole Helm. Published with license by Taylor & Francis.

This is an Open Access article distributed under the terms of the Creative Commons Attribution License (http://creativecommons.org/licenses/by/3.0/), which permits unrestricted use, distribution, and reproduction in any medium, provided the original work is properly cited. The moral rights of the named author(s) have been asserted.

Advances in complex regional pain syndrome (CRPS) suggest an association with maladaptive functional and structural neuroplastic change. Interventions that target maladaptive neuroplasticity may yield substantial improvements in pain and disability. This case presents a 10-year-old male with acute onset left lower extremity CRPS conservatively managed with multidisciplinary treatment. At initial assessment, patient reported significant pain (10/10), mobility and functional limitations, and impaired lower extremity left/right discrimination (accuracy left 80%, response time 3.1 s/accuracy right 0%, response time 0.6 s using Recognise app). Physical therapy intervention included brain based graded approach to movement including implicit motor imagery using the Recognise app. The app requires patient discrimination between images of left and right lower extremity limbs. Implicit motor imagery is thought to selectively activate the premotor cortex without activating primary motor areas. Over a one month period, data from the patient’s 185 uses of the Recognise app reveal gradual restoration of left and right discrimination (accuracy left 100%, response time 0.93 s/accuracy right 100%, response time 0.94 s) corresponding to significant reductions in pain and improved function. The patient subsequently reported increasing behavioural avoidance of pain-related cues (e.g., words and images) that triggered intense acute pain. Psychological intervention initially included graded exposure to pain-inducing cues; however, later focused on maintaining function using pain coping strategies. Pharmacological management included gabapentin and amitriptyline throughout. This case highlights the successful use of a graded approach to reduce overall threat of movement and allow body schema to be ‘exercised’ and re-organized in a safe context.

## The development of a national indigenous health research advisory committee on chronic pain

Brent Young^a^, Margot Latimer^b^, John Sylliboy^c^, Sharon Rudderham^d^, Maria Hudspith^e^, and Norman Buckley^f^

^a^Faculty of Medicine, Dalhousie University, Halifax, NS, Canada; ^b^Faculty of Health Professions, Dalhousie University, Halifax, NS; ^c^Centre for Pediatric Pain Research, IWK Health Centre, Halifax, NS, Canada; ^d^Health Director, Eskasoni Health Centre, NS, Canada; ^e^PainBC, Vancouver, BC, Canada; ^f^Department of Anesthesia, McMaster University, Hamilton, Ontario, Canada

**CONTACT** Brent Young brent.young@dal.ca

© 2017 Brent Young, Margot Latimer, John Sylliboy, Sharon Rudderham, Maria Hudspith, and Norman Buckley. Published with license by Taylor & Francis.

This is an Open Access article distributed under the terms of the Creative Commons Attribution License (http://creativecommons.org/licenses/by/3.0/), which permits unrestricted use, distribution, and reproduction in any medium, provided the original work is properly cited. The moral rights of the named author(s) have been asserted.

Background: Indigenous people in Canada suffer from the highest rates of pain but, as a patient population, are the least likely to receive treatment for it. Generally speaking, very few studies consider the unique perspectives of Indigenous people in the context of pain management. Indeed, researchers are eager to include Indigenous people in the research process, and Indigenous people want their stories heard; however, many Indigenous communities are hesitant to engage with external research groups. A legacy of disrespectful treatment from past research teams has led to an atmosphere of caution, uncertainty, and distrust. As such, there have been considerable efforts made towards reconciliation. In the absence of a one-size-fits-all approach, research teams must strive to establish more inclusive partnerships with Indigenous communities.

Innovation: The Aboriginal Children’s Hurt and Healing Initiative has emerged as a model for this type of collaboration. Using a two-eyed seeing approach, our team has engaged communities in the joint pursuit of knowledge surrounding pediatric pain management. Here, we discuss our most recent work in developing a national Indigenous Health Research Advisory Committee under the SPOR Chronic Pain Network. This committee is made up of stakeholders including patients, elders, academics, and health professionals who have been working to generate a body of Indigenous health research knowledge.

Relevance: This knowledge will be shared using multiple strategies with communities, academics, health professionals, policy makers, and the wider public with the goal of facilitating authentic partnerships and enhanced understanding between Indigenous communities and health researchers.

## Reflections on the development of a 6-week interdisciplinary chronic pain self-management program at Hotel Dieu Hospital in Kingston, Ontario

Emily Turnbull^a^, Kyle Vader^a^, Elizabeth Brown^a^, Judith Cox^a^, Tom Doulas^a^, Kerry Oulton^a^, Scott Duggan^b^, and Catherine Donnelly^c^

^a^Chronic Pain Clinic, Hotel Dieu Hospital, Kingston, Ontario, Canada; ^b^Anesthesiology and Perioperative Medicine, Queens University, Kingston, Ontario, Canada; ^c^School of Rehabilitation Therapy - Occupational Therapy Program, Queens University, Kingston, Ontario, Canada

**CONTACT** Emily Turnbull turnbule@hdh.kari.net

© 2017 Emily Turnbull, Kyle Vader, Elizabeth Brown, Judith Cox, Tom Doulas, Kerry Oulton, Scott Duggan, and Catherine Donnelly. Published with license by Taylor & Francis.

This is an Open Access article distributed under the terms of the Creative Commons Attribution License (http://creativecommons.org/licenses/by/3.0/), which permits unrestricted use, distribution, and reproduction in any medium, provided the original work is properly cited. The moral rights of the named author(s) have been asserted.

Background: The Chronic Pain Clinic at Hotel Dieu Hospital in Kingston, Ontario recently received funding to expand services to include an interdisciplinary self-management chronic pain program. The primary goals of the program are to support individuals in managing chronic pain and enhance engagement in everyday activities. As the only regional hospital-based interdisciplinary Chronic Pain Clinic, the secondary goal is to build capacity within the region with respect to pain-related services.

Approach: Working with the School of Rehabilitation Therapy at Queen’s University, a developmental evaluation was used to support both program development and lay the foundation for ongoing quality improvement. Three activities were conducted: 1) an environmental scan to identify current pain services in the region, 2) a scoping review to identify best evidence to inform the self-management program and, 3) a logic model to offer a program theory and visual depiction of program activities and outcomes.

Implementation: Based on results from the environmental scan, scoping review, and logic model, a 6-week self-management chronic pain program was developed. The first iteration has been implemented and was delivered by an interdisciplinary team including occupational therapy, physical therapy, social work, nursing, and medicine. The program is the first in the region and is offered to individuals across the South East Local Health Integration Network (SELHIN). The program includes both individual and group-based sessions emphasizing non-pharmacological approaches such as goal setting, pacing, stress management, and physical activity. The program will continue to be refined as a result of ongoing feedback throughout the developmental process.

## Management of sacro-pelvic cancer pain with retrograde intrathecal drug delivery

Uri Hochberg^a^ and Jordi Perez^a^

^a^The Alan Edwards Pain Management Unit/MUHC - The Cancer Pain Clinic, McGill University Health Centre (MUHC), Montreal, Quebec, Canada

**CONTACT** Uri Hochberg urihochberg@hotmail.com

© 2017 Uri Hochberg and Jordi Perez. Published with license by Taylor & Francis.

This is an Open Access article distributed under the terms of the Creative Commons Attribution License (http://creativecommons.org/licenses/by/3.0/), which permits unrestricted use, distribution, and reproduction in any medium, provided the original work is properly cited. The moral rights of the named author(s) have been asserted.

Management of sacro-pelvic cancer pain is particularly challenging with medication because of its particular sensory innervation. Spinal drug delivery is an analgesic option when conventional medical fails but a key factor to successful spinal analgesia is catheter placement close to the source of the pain.

We describe 3 cancer pain cases successfully managed with retrograde intrathecal delivery.

**Patient 1:** 84 y/o male presenting with sacral and leg pain diagnosed with L3-L4 fractures and epidural extension of metastatic multiple myeloma

**Patient 2:** 78 y/o male presenting with buttock and leg pain diagnosed with metastatic prostate cancer invading S1.

Both patients were bedridden associating severe pain with ambulation and sitting.

**Patient 3:** 67 y/o female presenting with genital pain preventing from sitting diagnosed with locally advanced vulvar carcinoma.

Patients were admitted in palliative care for symptom control, conventional analgesia failed or associated neurotoxicity, percutaneous blocks were contraindicated thus spinal drug delivery was considered.

Patients underwent intrathecal catheter placement inserted through L2-L3 and advanced in a retrograde fashion towards the sacrum. Catheters were tunneled and connected to a port-a-cath. An external infusion with Fentanyl 2 µg/ml + Bupivacaine 0.6mg/ml was initiated.

Satisfactory analgesia was achieved rapidly with low volumes (range 1 to 3 ml/h). Functional improvement, reduction of PRN consumption and neurotoxicity were observed without associating neurological deficits. The regimen lasted 19 days on average (range 10-25 days). All patients died of their underlying cancers comfortably.

The advantages and disadvantages and a literature review of this approach will be presented.

## Advancing research and clinical care in neuropathic pain after spinal cord injury: Recommendations from a Canadian summit

Eldon Loh^a^, Stacey Guy^b^, Tara Jeji^c^, Phalgun Joshi^d^, Vanessa Noonan^d^, and Keith Hayes^c^

^a^Physical Medicine and Rehabilitation,Western University, London, ON, Canada; ^b^Parkwood Institute Research, London, ON, Canada; ^c^Ontario Neurotrauma Foundation, Toronto, ON, Canada; ^d^Rick Hansen Institute, Vancouver, BC, Canada

**CONTACT** Eldon Loh eldon.loh@sjhc.london.on.ca

© 2017 Eldon Loh, Stacey Guy, Tara Jeji, Phalgun Joshi, Vanessa Noonan, and Keith Hayes. Published with license by Taylor & Francis.

This is an Open Access article distributed under the terms of the Creative Commons Attribution License (http://creativecommons.org/licenses/by/3.0/), which permits unrestricted use, distribution, and reproduction in any medium, provided the original work is properly cited. The moral rights of the named author(s) have been asserted.

Due to the prevalence and negative effects of neuropathic pain (NP) after spinal cord injury (SCI), a key strategic priority for the Ontario Neurotrauma Foundation (ONF) and the Rick Hansen Institute (RHI) is the improvement of NP management after SCI. To achieve this goal, a transdisciplinary panel of eighteen key stakeholders from across Canada participated in a National Summit on November 4, 2016 to develop a strategic plan.

The specific aims of the summit were to 1) describe the current state of the field, 2) develop a shared long-term vision of results for ONF and RHI to achieve within 5–10 years, and 3) identify steps for moving forward.

The panel identified numerous strengths within the field nationally, including the SCI Research Evidence (SCIRE) website, the CanPainSCI Clinical Practice Guidelines (CPG), the rehabilitation E-scan, the RHI SCI Registry, and the SCI Knowledge Mobilization Network. Identified gaps included a limited evidence base, absence of an integrated national strategy, limited connections across the care continuum, limited consumer involvement, and lack of implementation of the existing CanPainSCI CPG. The long-term vision proposed by the summit panel recommends the establishment of a collaborative clinical and research network, standardization through implementation, standardized evaluation of impact, empowerment through education, advocacy, and the direction of resources to innovative solutions.

Moving forward, a working group will prioritize specific items within the long-term vision to provide the greatest clinical impact in a reasonable timeframe. This group will be convened to address the summit recommendations in early 2017.

## A five year review of our home nerve block program. Having patients recover comfortably at home

Paula Hammond^a^, Lynn Langille^a^, and Jennifer Szerb^b^

^a^Acute Pain Service, Nova Scotia Health Authority (NSHA), Halifax, NS, Canada; ^b^Anesthesia Dalhousie University, Halifax, NS, Canada

**CONTACT** Paula Hammond paula.hammond@nshealth.ca

© 2017 Paula Hammond, Lynn Langille, and Jennifer Szerb. Published with license by Taylor & Francis.

This is an Open Access article distributed under the terms of the Creative Commons Attribution License (http://creativecommons.org/licenses/by/3.0/), which permits unrestricted use, distribution, and reproduction in any medium, provided the original work is properly cited. The moral rights of the named author(s) have been asserted.

Introduction/Aim: The home nerve block program is aimed at improving postoperative analgesia for patients by allowing them to receive continuous peripheral nerve block infusions at home following discharge from hospital. A home nerve block infusion provides the added benefits of an earlier discharge from hospital and potential for less opioid use. A trial was conducted from February to April of 2011 with 12 patients post ankle fusion surgery. Post trial we were given the approval to offer home nerve block as an ongoing basis for ankle surgeries. The program began in April 2012 and expanded in April 2013 to include other orthopedic surgeries such as total shoulder.

Methods: Identification of patients and obtain consent

1. Provide patient education and assess patient needs
2. Connect to portable infusor on day of discharge or in PACU
3. Telephone follow up daily by a member of APS team until nerve block is removed by patient

Discussion/Conclusions: During the five years the program has run we were able to capture quantitative data that demonstrates the breadth of our program (type of surgeries, patient satisfaction and opioid use). The strength of our program was to standardize patient teaching kit/equipment and provide adequate follow up and patient access to APS physician 24/7. Some of the challenges we have faced include: discharging to rural areas, day of surgery decisions by patients, titration of infusions before discharge to aim for motor function without pain (in presence of circumferential cast) and physician remuneration.

## A novel application and evaluation of mindfulness-based cognitive therapy for chronic pain

Denise Paneduro, Kethmini Amarasinghe, Sally Chung, Ted Robinson, Angie Kingma, Kareena Gurbaxani, Marilyn Galonski, and Allan Gordon

Wasser Pain Management Centre, Sinai Health System, Toronto, ON, Canada

**CONTACT** Denise Paneduro Denise.Paneduro@sinaihealthsystem.ca

© 2017 Denise Paneduro, Kethmini Amarasinghe, Sally Chung, Ted Robinson, Angie Kingma, Kareena Gurbaxani, Marilyn Galonski, and Allan Gordon. Published with license by Taylor & Francis.

This is an Open Access article distributed under the terms of the Creative Commons Attribution License (http://creativecommons.org/licenses/by/3.0/), which permits unrestricted use, distribution, and reproduction in any medium, provided the original work is properly cited. The moral rights of the named author(s) have been asserted.

Introduction/Aim: To evaluate the effectiveness of a novel application of Mindfulness-Based Cognitive Therapy (MBCT) for improving physical, psychological, and social health associated with chronic pain and to explore the role of adult attachment styles on treatment outcomes.

Methods: Twenty-nine adults with chronic pain participated in 1 of two 8-week MBCT group programs offered consecutively. Outcomes were assessed with self-report standardized questionnaires prior to and following treatment and included secure, avoidant, and anxious attachment styles, pain intensity, pain disability, pain catastrophizing, depression, anxiety, stress, mindfulness, and perceived social support.

Results: Paired t-tests revealed significant decreases from pre-treatment to post-treatment in pain intensity, pain disability, pain catastrophizing, and depression, and an increase in mindfulness. Following MBCT treatment, participants with a secure attachment style showed significant increases in feelings of security and individuals with an avoidant attachment style demonstrated decreases in avoidance. Significant negative associations were obtained between security and emotional distress such that participants with increased security had decreased levels of depression and stress. Significant positive associations were found between secure attachment and perceived social support and between avoidant attachment and pain catastrophizing.

Discussion/Conclusions: These findings demonstrate reduced pain, psychological distress, and improvements in adult attachment styles following MBCT. Associations observed between attachment styles and psychosocial outcomes highlights the importance of considering individual vulnerability factors like attachment as part of a comprehensive model of chronic pain. Future controlled trials are needed to confirm these findings and further explore the role of attachment in pain onset, maintenance, and recovery.

## Integrating chronic pain services into the temporomandibular surgical care pathway

Jane Burns^a^, Jaclyn Ricci^b^, Penny Dooks^c^, Victoria Antoniu^d^, Farah Khan Choudhry^e^, Brian Rittenberg^f^, Leah Pink^a^, Allan Gordon^g^, Denise Paneduro^h^, and Marilyn Galonski^i^

^a^Wasser Pain Management Center, Nursing, Sinai Health System, Toronto, Ontario, Canada; ^b^Acute Pain Service, Nursing, Sinai Health System, Toronto, Ontario, Canada; ^c^Nursing, Sinai Health System, Toronto, Ontario, Canada; ^d^Quality, Sinai Health System, Toronto, Ontario, Canada; ^e^Nursing Administration, Sinai Health System, Toronto, Ontario, Canada; ^f^Dentistry, Sinai Health System, Toronto, Ontario, Canada; ^g^Wasser Pain Management Center, Nursing Administration, Sinai Health System, Toronto, Ontario, Canada; ^h^Wasser Pain Management Center, Medicine, Sinai Health System, Toronto, Ontario, Canada; ^i^Wasser Pain Management Center, Sinai Health System, Toronto, Ontario, Canada

**CONTACT** Jane Burns jane.burns@sinaihealthsystem.ca

© 2017 Jane Burns, Jaclyn Ricci, Penny Dooks, Victoria Antoniu, Farah Khan Choudhry, Brian Rittenberg, Leah Pink, Allan Gordon, Denise Paneduro, and Marilyn Galonski. Published with license by Taylor & Francis

This is an Open Access article distributed under the terms of the Creative Commons Attribution License (http://creativecommons.org/licenses/by/3.0/), which permits unrestricted use, distribution, and reproduction in any medium, provided the original work is properly cited. The moral rights of the named author(s) have been asserted.

Hospitals across Canada are continuously looking for ways to save health care costs and safely decrease inpatient hospital stays. A primary reason for an increase in length of stay in the temporomandibular joint (TMJ) replacement population at Sinai Health System is suboptimal management of pain. Mount Sinai Hospital’s Center of Excellence initiative was developed following an examination of length of stay (LOS) and case costing data. TMJ replacement was identified as an ideal patient population for introducing the chronic pain service early into a care pathway to help better manage chronic pain prior to surgery, following joint repair, and into follow-up.

An interdisciplinary team was established representing health care providers across the TMJ care continuum with the overarching goal of integrating primary care and hospital care. The team agreed on the following objectives: 1) to ensure that TMJ patients with chronic pain are identified as early as possible in the care pathway; 2) identify the best place in the care pathway to provide integrated chronic pain services; 3) develop the necessary tools and processes to ensure success for the patient experiencing chronic pain and awaiting surgery; and 4) evaluate each step of the pathway to optimize patient care, patient experience, decrease LOS and manage acute and acute on chronic pain. This initiative is the first of its kind involving primary care and chronic pain services early in the care pathway and will serve as the template for other complex pain patient populations in future.

## A year in the development of a new interdisciplinary pediatric chronic pain program: Opportunities, initiatives, and challenges

Kim Edwards, Nez Elik, and C. Meghan McMurtry, and other members of the Pediatric Chronic Pain Program at McMaster Children’s Hospital 0000-0002-3278-1169

Pediatric Chronic Pain Program, McMaster Children’s Hospital, Hamilton, Ontario, Canada

**CONTACT** C. Meghan McMurtry cmcmurtr@uoguelph.ca

© 2017 Kim Edwards, Nez Elik, C. Meghan McMurtry, and other members of the Pediatric Chronic Pain Program at McMaster Children’s Hospital. Published with license by Taylor & Francis.

This is an Open Access article distributed under the terms of the Creative Commons Attribution License (http://creativecommons.org/licenses/by/3.0/), which permits unrestricted use, distribution, and reproduction in any medium, provided the original work is properly cited. The moral rights of the named author(s) have been asserted.

“*…The field of pediatric pain medicine has demonstrated the benefits of interdisciplinary collaboration more than any other endeavour*” (Law, Palermo, & Walco, 2013). Recently, the Ontario Ministry of Health and Long-term Care announced the funding of specialty pediatric chronic pain programs in several children’s hospitals across the province, including McMaster Children’s Hospital. The Pediatric Chronic Pain Program includes Physicians (Pediatricians, Psychiatrist, Anesthesio-logist), Psychologists, Child Life Specialist, Registered Nurse, Nurse Practitioner, Occupational Therapist, Physiotherapist, Social Workers, Pharmacist, and Clinical Manager. The purpose of this poster is to highlight new opportunities and initiatives within our clinic, including the development and facilitation of standardized interdisciplinary assessment and treatment processes, research database and program evaluation integrated with clinical work, development of new group therapies (e.g., a 10-week Psychoeducation Rise Above Pain Group for both parents and youth; a 5-week Parenting Youth with Chronic Pain Group; a 5-week Exercise Group for youth with chronic pain). Challenges in developing a new clinic/new programs and providing care to complex families (e.g., professional roles and competencies, diagnostic discrepancies) will be discussed. Implications for program development in new and established clinics will be highlighted.

## Removing the barriers to pain management: A novel, collaborative model of service delivery between academic hospital chronic pain centers

Tania Di Renna^a^ and Laura Pus^b^

^a^Anesthesiology, Women’s College Hospital, Toronto, ON, Canada; ^b^Toronto Academic Pain Medicine Institute, Women’s College Hospital, Toronto, ON, Canada

**CONTACT** Tania Di Renna tania.direnna@wchospital.ca

© 2017 Tania Di Renna and Laura Pus. Published with license by Taylor & Francis.

This is an Open Access article distributed under the terms of the Creative Commons Attribution License (http://creativecommons.org/licenses/by/3.0/), which permits unrestricted use, distribution, and reproduction in any medium, provided the original work is properly cited. The moral rights of the named author(s) have been asserted.

**Toronto Academic Pain Medicine Institute (TAPMI)** is a comprehensive virtual network of pain management services in downtown Toronto. It is a partnership whereby all academic pain management hospitals — Centre for Addiction and Mental Health, Sinai Health System, St. Michael’s Hospital, University Health Network and Women’s College Hospital — work together to provide seamless care for Ontario’s chronic pain patients via a **centralized referral network**. TAPMI is uniquely positioned to offer patients a wide variety of **interdisciplinary provider** options including (but not limited to) physiotherapy, medical treatments, cognitive behavioral therapy, chiropractic services, occupational therapy that are **fully funded by OHIP**. Patients are referred through a simplified central intake process where they are triaged to the most appropriate academic pain clinic. During their wait to be assessed by the pain clinic, patients are offered pain education, access to self-management programs, readiness for change assessment tools, psychology and physiotherapy assessments for group programs. Once the patient has completed the program, primary health care providers are offered access to discharge clinic services where academic pain specialists see the patients with the family MD for discharge planning in the comfort of their own primary practice. Primary care providers are offered access through eConsultation and telemedicine if the patient requires reconsultation to TAPMI This approach is novel in that it offers fully funded programs that are accessible during the waiting period to see an academic pain clinic. The program also addresses the need for coordinated discharge planning and support for primary care providers.

